# Effect of heat and pectinase maceration on phenolic compounds and physicochemical quality of *Strychnos cocculoides* juice

**DOI:** 10.1371/journal.pone.0202415

**Published:** 2018-08-17

**Authors:** Ruth T. Ngadze, Ruud Verkerk, Loveness K. Nyanga, Vincenzo Fogliano, Rosalia Ferracane, Antonio D. Troise, Anita R. Linnemann

**Affiliations:** 1 Department of Food Science and Technology, Chinhoyi University of Technology, Chinhoyi, Zimbabwe; 2 Food Quality and Design, Wageningen University & Research, Wageningen, The Netherlands; 3 Institute of Food, Nutritional & Family Sciences, University of Zimbabwe, Harare, Zimbabwe; 4 Department of Agricultural Sciences University of Naples “Federico II”, Portici (NA), Italy; EMBRAPA Agroindustrial Tropical, BRAZIL

## Abstract

*Strychnos cocculoides* fruit is an important food source for rural populations in Zimbabwe in times of scarcity. Its thick pulp tightly adheres to its seeds, causing pulp extraction constraints and waste during processing, leading to underutilisation. Therefore, pectinase maceration combined with heat treatments was studied to improve juice yield and juice quality. Metabolite profiling according to the heat map, FancyTile chromatic scale approach and phenolic compound content were used to compare the identified compounds. Prior to treatments, 16 known phenolic compounds, predominantly belonging to the phenolic acids, flavonoids and iridoid glucoside classes, were tentatively characterized for the first time in *S*. *cocculoides* using High Resolution Mass Spectrometry and LC/MS/MS. Overall, results showed that enzymatic treatments increased pulp yield (by 26%), physicochemical quality (38% increase in juice clarity), content of phenolic compounds (predominantly kaempferol, quercetin, caffeic acid, protocatechuic acid, iridoids) and antioxidant activity.The improved extraction of *S*. *cocculoides* pulp increases juice yield as well as juice quality by supplying larger amounts of phenolic compounds that have potential health benefits and act as dietary sources of antioxidants for the prevention of diseases caused by oxidative stress.

## Introduction

Monkey orange species (e.g. *Strychnos cocculoides*) possess viscous flesh tightly adhering to hard seeds at 1:2 w/w ratio pulp and seed fresh weight (FW), hampering pulp extraction by simple pressing. The fruit is consumed fresh or after pulp extraction [1]. The limited seed–pulp separation reduces pulp yield with a subsequent increase in waste of nutritious food. Generally three extraction methods are used to increase the yield of pulp and juice viz., hot, cold or enzymatic pre-treatments [2]. Pre-treatment with pectinolytic enzymes has been used successfully for fruit pulp since the 1930s to soften tissues, ease seed/pulp separation and increase juice yield [3]. Enzymes hydrolyse glucoside bonds of the main chains of polygalacturonic acid in cell walls for increased recovery of soluble fruit components and sensory quality [4]. An appropriate enzymatic maceration treatment for the *S*. *cocculoides* matrix is expected to improve pulp yield and juice quality.

*S*. *cocculoides* fruit features nutrients important to human health, such as minerals and vitamin C [5]. Moreover, the fruit contains bioactive phenolic compounds (PCs), occurring as different classes of secondary metabolites, like many other fruits. The PCs reportedly have health benefits beyond basic nutrition and contribute to antioxidant activity (AOA) within the human body[6]. PCs also contribute to fruit characteristics such as colour, flavour, bitterness, astringency and, together with polysaccharides, add to chemical stability and sensory perception [7]. Other phytochemicals such as iridoid glycosides (IGs), which have potential biological activities, [8] were reported in *S*. *cocculoides* bark [9].

*S*. *cocculoides* trees thrive during the dry season and remain dormant when water is unavailable. Abiotic stress can elicit the presence and lead to high concentrations of particular PCs, [10] providing a potential source of healthy and nutritious fruits in drought periods. *S*. *cocculoides* products are underutilized, warranting technological solutions to this challenge. The current study identified the PCs and assessed the effect of maceration using a novel representation that combines a heat map, hierarchical clustering and FancyTile approach. The FancyTile approach uses a targeted metabolite profiling of different class metabolites by High Resolution Mass Spectrometry (HRMS) with a chromatic representation of the variations in different samples [11]. This study reports PCs in *S*. *cocculoides* and proposes processes to improve pulp yield and health beneficial compounds.

## Materials and methods

### Standards and chemicals

Cynarine, caffeic acid (CA), chlorogenic acid, protocatechuic acid (PCA), rutin, quercetin, myricetin, kaempferol, naringenin 7-*O*-glucoside, naringenin and catechin standards were purchased from SigmaAldrich (Italy), and loganic acid from Extrasynthese (France). Commercial pectinase enzymes from *Aspergillus niger* were obtained from SigmaAldrich (Germany).

### Enzyme treatment and pulp extraction

**Fig 1:**
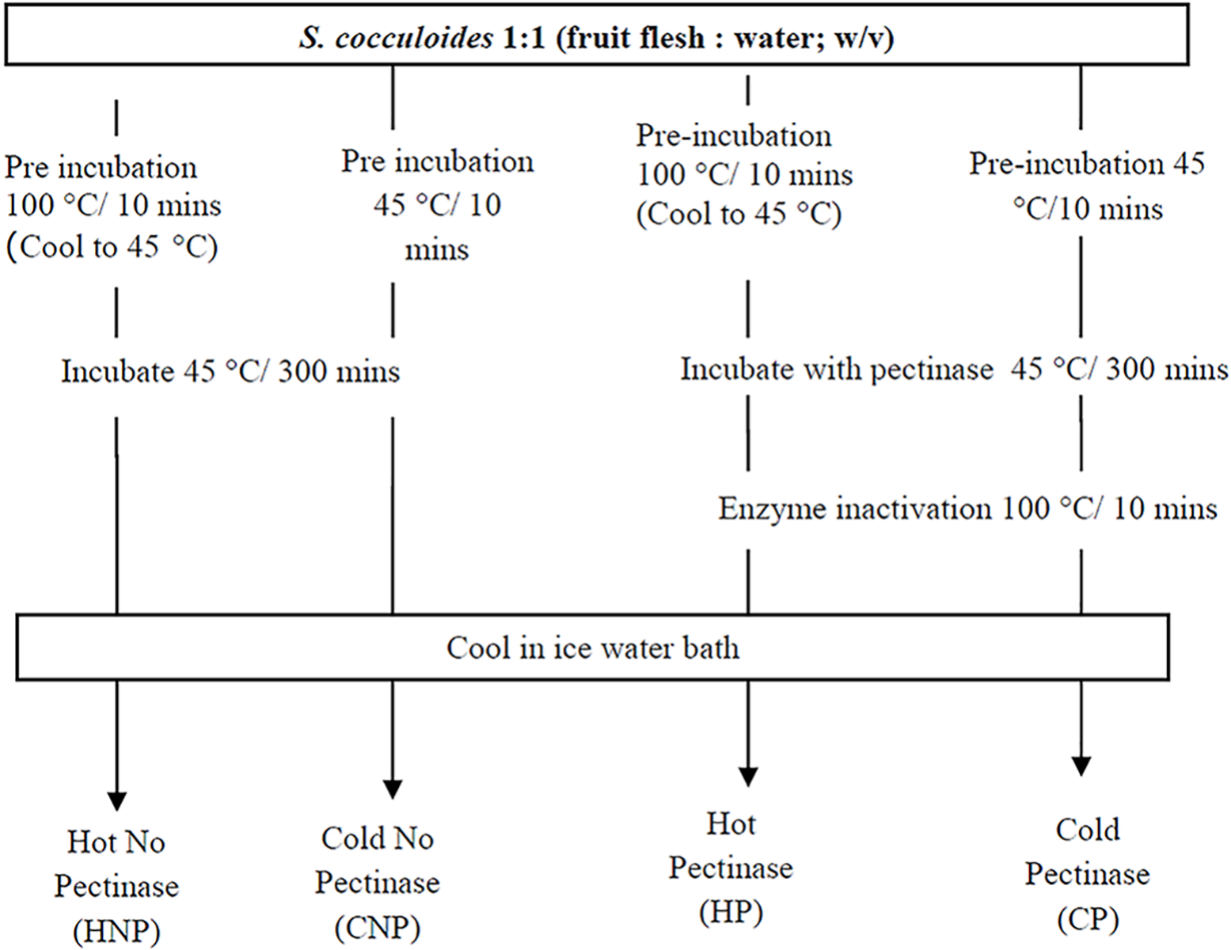
*Strychnos cocculoides* fruit treatments (HNP, CNP,HP and CP). HNP: Hot water extraction without pectinase, CNP: Cold water extraction without pectinase, HP: Hot water extraction with pectinase, CP: Cold water extraction with pectinase.

Ripe *Strychnos cocculoides (S*. *cocculoides)* fruits (i.e. having a bright orange colour) without any visual defects were collected from Lower Gwelo, Zimbabwe, and transported at 4°C to Wageningen University & Research (The Netherlands). Fruits were shelled by hammering, flesh blended and stored at -20°C until further analysis. Enzymatic experiments were performed on the pulp with pectinase at 0.5 to 2% w/v. Scheme of enzymatic and non-enzymatic treatments is shown in Fig 1 for HNP: Hot water extraction without pectinase, CNP: Cold water extraction without pectinase, HP: Hot water extraction with pectinase, CP: Cold water extraction with pectinase. Pulp yield was determined by: $Y=\left(\frac{A}{B}\right)X100$. Where Y is the % pulp yield, A is detached pulp weight (g) and B is initial fruit weight (g). The variables that produced the highest pulp yield and dry matter were used as optimal treatments. Fig 1 depicts the experimental set-up. The resultant pulp/juice mixture was further analysed, starting with centrifugation at 4700 rpm for 15 min to remove coarse pulp particles. The supernatant was considered as the obtained amount of juice. Dry matter was determined as described in AOAC [12].

### Physicochemical properties

The pH was measured with a pH meter (pH1002 VWR phenomenal). Soluble solids (°Brix) were determined with a refractometer. Clarity was measured as the % transmittance at 660 nm using a Cary 50 Bio UV-vis spectrophotometer with distilled water as a blank [13]. Colour measurements, i.e. CieLab coordinates (L*, a*, b*), were directly read with a Hunter ColorFex LAB (Escolab Netherlands) apparatus, calibrated with a black, green and white tile. Colour differences (delta) were expressed as ΔE* = [(ΔL*^2^) + (Δa*^2^) + (Δb*^2^)]^1/2^ to have one colour value [14]. The sample with the lightest colour was used as reference.

### Sugar analysis

Samples were centrifuged at 4700 rpm for 15 min at 20°C, after which 1 ml of residue was diluted up to 20 times with distilled water. External standards for sucrose, glucose and fructose in the range of 2.5–0.078 mg/ml were used for extract quantification. Standards and extracts were filtered through a 0.45 μm filter for high performance liquid chromatography (HPLC) analysis. Individual sugars were separated by HLPC using a Thermo Separation Products model with P-2000 Pump and ELSD-2100 polymer lab detector. Separation was conducted on a Grace prevail carbohydrates column, 5μm, (250 mm x 4.6 mm) with an evaporator temperature of 90°C, nebuliser temperature of 50°C and gas 1.6 arbitrary units. Running time was 30 min with a flow rate of 0.8 ml/min on isocratic 75/25% acetonitrile/water. Extract peaks were identified by comparing retention times (RT) with sugars standards.

### Characterization of phenolic compounds

Freeze-dried samples (1.0 g) were dissolved in 20 mL of methanol/water (70:30, *v/v*) and homogenized with an Ultra-Turrax T25 digital (Ika, Germany) for 2 min, ultrasonically extracted for 30 min, then centrifuged at 14800 rpm for 10 min. The supernatants were analysed by HRMS. LC-MS data were acquired on an Accela U-HPLC system coupled to an Exactive mass spectrometer (Thermo Fisher Scientific, San Jose, CA). Chromatographic conditions were as described by Zhu, Chen [15] with some modifications: a Kinetex 2.6 μm, C18 100 A column (100 mm × 2.1 mm) (Phenomenex, Torrance, CA) thermostated at 40°C was used with a mobile phase of 0.1% formic acid/water (A) and 0.1% formic acid/acetonitrile (B). Gradient elution was linearly programmed as follows: 2% B (0.5 min), 2–10% B (0.5–6) min, 10–33% B (6–20 min), 33–90% B (20–23 min). Flow rate was set at 300 μL/min. The U-HPLC was directly interfaced to an Exactive Orbitrap MS equipped with a heated electrospray interface (HESI-II). Acquisition was performed in positive and negative ionization modes (*m/z* 100–1500). Chromatographic data acquisition and peak integration were performed using Xcalibur software (Thermo Fisher Scientific, San Jose, USA). Identification was by comparison of RT and MS data with those of reference standards. Other compounds were tentatively assigned using exact mass up to the fifth decimal digit with a mass tolerance ± 5 ppm. Calibration curves of flavonoids were constructed in the linearity range: 10–5000 ng/mL, phenolic acids and loganic acid: 100–5000 ng/mL. All IGs were expressed as equivalents of loganic acid, kelampayoside A as equivalents of rutin, and 2-hydroxy-3-*O-β-*D-glucopyranosyl-benzoic acid as equivalents of PCA. Identification of IGs was based on MS/MS analysis performed on an API 3000 triple quadrupole mass spectrometer (Applied Biosystems, Canada) and comparing fragmentation patterns with literature data.

### Antioxidant activity determination

The AOA was assessed by the free-radical scavenging effect on the 2, 2-diphenyl-1-picrylhydrazyl (DPPH) radical as described by Brand-Williams, Cuvelier [16] with some modification. Pulp extract was mixed with 100% aqueous methanol, the suspension incubated for 30 min at room temperature while vortexing at 1620 rpm with a Heidolph multi tube mixer, then ultrasonically extracted for 30 min at 35°C and finally centrifuged for 10 min at 4000 rpm at 4°C. Further analysis were as described by Hiwilepo-van Hal, Bille [17].

### Statistical analysis

All results are given as means ± standard deviation of three fully independent determinations of each parameter. Significant differences between means were determined by one-way analysis of variance with the Tukey test. Relationships among variables were determined with Pearson correlation coefficients; differences were considered significant at p < 0.05. The Statistical Package for Social Sciences (SPSS v23.0) software was used.

### Multivariate analysis

Multivariate data analysis was conducted in three steps according to the concentration of the polyphenols in the samples. The heatmap was calculated using the function OMICs in XLSTAT environment (Addinsoft, NY v 2014.5.03). Next dendrograms were constructed by the agglomerative hierarchical clustering algorithm proposed by Ward [18]. The method aggregates two groups to limit within-group inertia as much as possible to keep clusters homogeneous. Finally, according to the clusters, a FancyTiles chromatic scale was composed as described by Troise, Ferracane [11] by considering a fixed range among the treatments. A summary of the RT, chemical formulae, selected ion and theoretical masses of the PCs are reported.

## Results and discussion

### Pulp yield

Maximum pulp yield and dry matter were obtained at 0.5% (w/v) pectinase and incubation for 300 min; a further increase in pectinase concentration had no significant effect on pulp yield (preliminary experiments, data not shown). The pulp yields of cold-no-pectinase (CNP) and hot-no-pectinase (HNP) were 59 and 60%, respectively, while hot-pectinase (HP) and cold-pectinase gave an 85% pulp yield and 12.5% dry matter. With the addition of pectinase to *S*. *cocculoides*, the tissue of the pulp disintegrated and detached better from the seed. Enzyme treatment proved to be advantageous for high pulp recovery, hence reducing processing wastage. Pectinase is a crude enzyme preparation having multiple enzyme activities: polygalacturonase, pectate lyases and pectin methylesterase, which enhances juice yield and extraction [4, 19]. Heating prior to enzymatic extraction further increases extract recovery by depolymerizing lignin components and softening fruit tissue [2].

### Effect of enzymatic treatments on physicochemical quality

Table 1 presents ᵒBrix, pH, clarity and colour data after pulp maceration. HP treatment gave the highest ᵒBrix value (10.1) and the lowest pH (3.3). Enzyme treatments resulted in a significantly higher clarity of the juice than the non-enzyme treatments. The small difference between enzyme-treated and non-enzyme treated samples, namely ΔE* < 1.8 and ΔE* < 0.5 between CP and HP, respectively, made colour perceptually insignificant. High ᵒBrix after enzyme extraction was due to increased tissue breakdown thus releasing sugars, which contributed to soluble solids. Low pH due to HP is explained by the release of carboxylic acids and galacturonic acid during enzyme treatment. High amounts of carboxylic acids, namely succinic and citric acid, were found in edible *Strychnos spinosa (S*. *spinosa)* [20]. A relatively lower pH positively impacts the keeping quality of the juice as a mild preservative and sensory quality by maintaining sugar/acid ratios. Pectinase hydrolyses pectin, causing pectin-protein complexes to aggregate into larger particles that settle and improve juice clarity; [21] our data corroborate these findings. Large pulp fragments in non-enzyme treated samples were removed by centrifugation, but smaller fragments, insoluble calcium pectate and tissues [21] remained, leading to poor clarity of juice.

**Table 1:** Physicochemical properties of *Strychnos cocculoides* juice.

Treatment	Total soluble solids (°Brix)	pH	Clarity (% transmission at 660 nm)	Colour		
				L*	a*	b*
CNP	9.3 ± 0.23^b^	3.6 ± 0.00^a^	56.1 ± 2.33^b^	36.7	-1.11	7.76
HNP	9.6 ± 0.35^a b^	3.43 ± 0.58^b^	53.0 ± 0.23^c^	36.5	-1.01	7.71
HP	10.1 ± 0.12^a^	3.33 ± 0.1^c^	92.1 ± 0.18^a^	37.9	-1.32	6.43
CP	9.9 ± 0.27^a b^	3.53 ± 0.58^a^	90.8 ± 0.13^a^	37.6	-1.25	6.03

Different letters in the same column indicate significant differences at p<0.05. Data are reported as mean ± standard deviation (n = 3). L*, a*, b* CIE colour values showing colour change of *Strychnos cocculoides* juice per treatment

CNP: Cold water extraction without pectinase, HNP: Hot water extraction without pectinase, HP: Hot water extraction with pectinase, CP: Cold water extraction with pectinase.

An orange-brown colour is a preferred characteristic of *S*. *cocculoides* food products [22]. Both enzymatic and non-enzymatic maceration treatments gave this feature. In cold extractions the colour can be related to enzymatic browning by naturally occurring polyphenol oxidases in the fruit, which catalyse phenol oxidation to form quinones which then polymerize to form melanoidins [21]. The expectation was that enzyme bleaching by hot treatments would result in a lighter product through inactivation of the endogenous enzymes by the pre incubation step (100°C for 10 min). However, this was not the case. During hot extractions other mechanisms such as non-enzymatic browning would have occurred such as PCs metal ion complexes (mainly Fe and Cu), ascorbic acid degradation and/or Maillard reactions, [23] resulting in the mixed effects on colour that occurred. These insignificant differences are advantageous as they apparently do not cause any undesirable effects to the colour of the product. However, unwanted flavour changes may arise from the additive effects of pre-treatment incubation and inactivation of pectinase after treatment, leading to flavour losses or development of cooked flavours, effects that need to be investigated.

### Effect of enzymatic treatment on individual sugars

Soluble sugars identified post maceration were fructose: 200–240 g kg^-1^ DW and glucose: 170–250 g kg^-1^ DW. Sucrose was only detected in the untreated fruit juice (44 g kg^-1^ DW). It was unquantifiable by the assay used, mainly due to the maceration dilution (1:1) and assay dilution (x 20). Though not significantly different, higher fructose and glucose contents were obtained by cold extractions than by hot extractions. Sitrit, Loison [24] reported a 2-fold increase in the conversion of sucrose to glucose and fructose during ripening of *S*. *spinosa*. The low sucrose content from our findings showed that sucrose was converted into its monomeric sugars during ripening and storage of *S*. *cocculoides*, a characteristic that is common in climacteric fruits [25]. High sugar levels after cold extraction are attributed to soluble or cell wall bound invertase (optimum temperature 40–50°C), [26] enhanced by the lower pre-incubation temperatures. The sugar results provide useful information for further study on the use of macerated *S*. *cocculoides* as a source of rapid energy, sensory appeal, ingredient for confectionery, juice and candy sweetening.

### Effect of enzymatic treatments on the content of phenolic compounds

**Fig 2:**
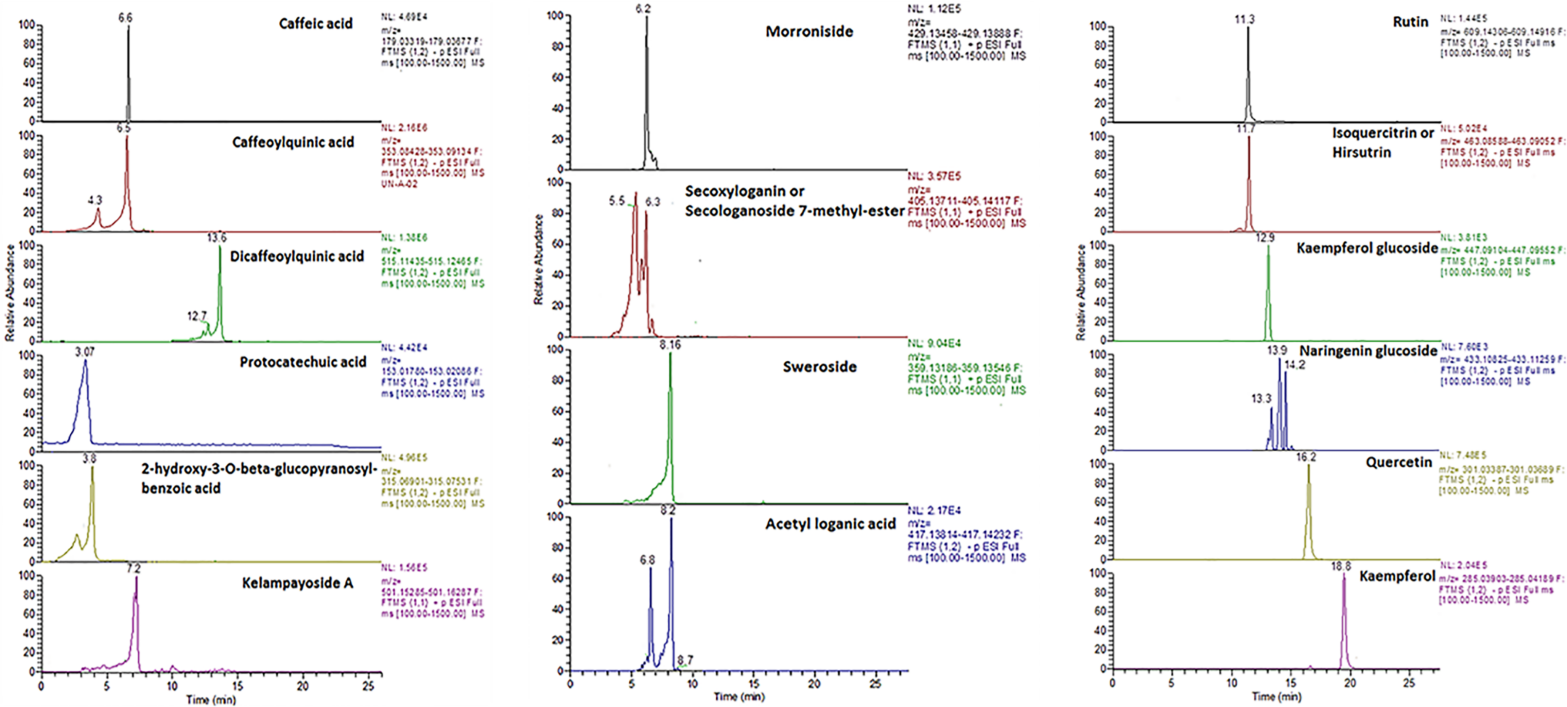
Extracted ion chromatograms of a: phenolic acids and kelampayoside A (phenolic apioglucoside), b: IGs and c: flavonoids in a representative extract of *Strychnos cocculoides* juice.

Sixteen PCs were tentatively identified in *S*. *cocculoides* juice by HRMS and iridoids were confirmed by tandem mass spectrometry, Fig 2. This is the first complete description of the polyphenol profile of the edible part of *S*. *cocculoides*.

#### Phenolic acids

**Fig 3:**
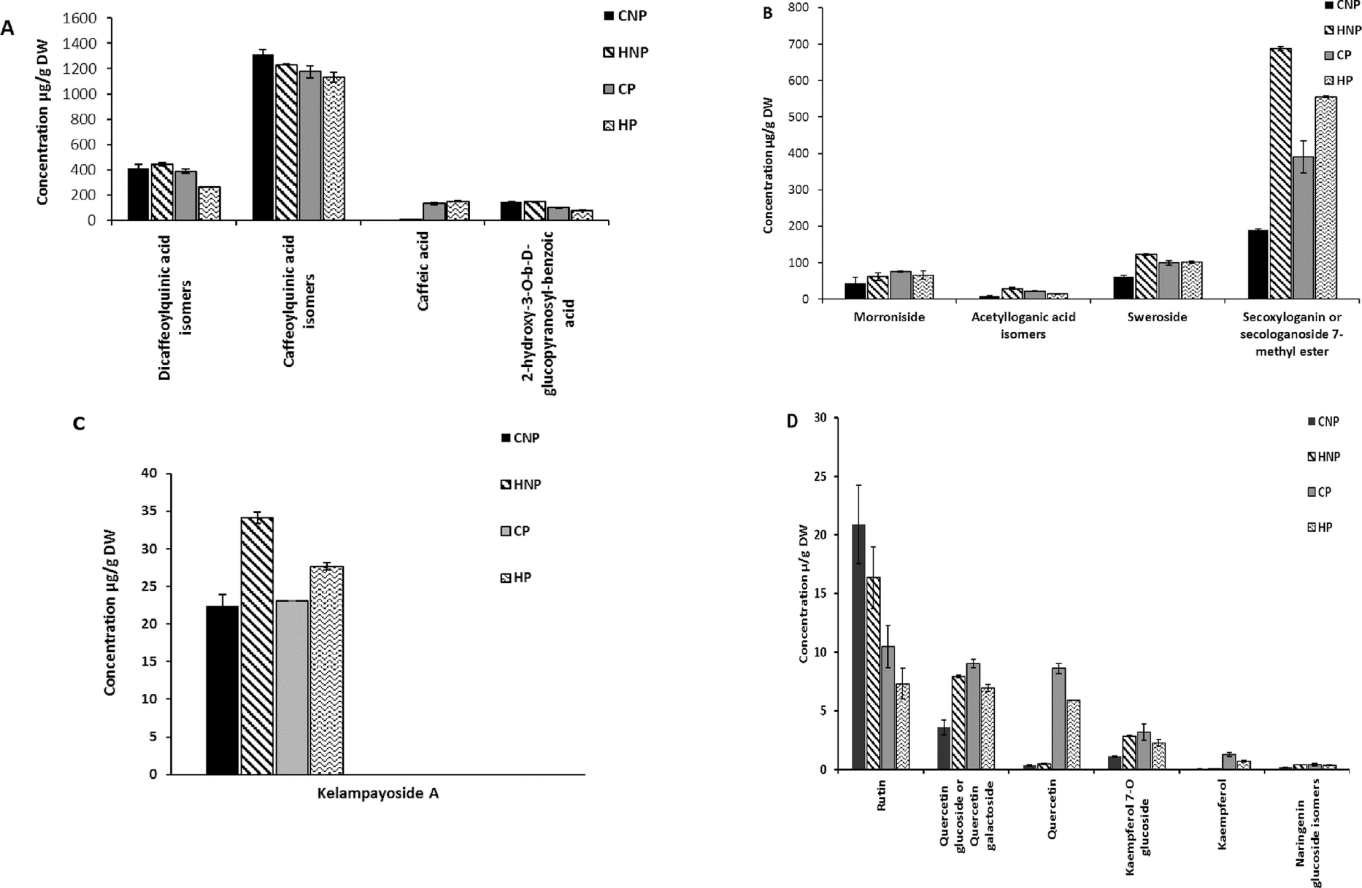
Phenolic compounds content of A: Phenolic acids, B: IGs, C: Kelampayoside A, D: Flavonoids, of *Strychnos cocculoides* pulp. Data are expressed as μg/ g of dry weight ± standard deviation with n = 3.

Pectinase treatments gave a slightly reduced content of caffeoylquinic acid, though not significantly different (p>0.05) from that of the treatments without pectinase (Fig 3A). Enzyme and heat treatments decreased caffeoylquinic acid content, complementary to an increase in CA (*R* = -0.893). Caffeic acid is uncommonly found as a free acid in unprocessed plant material, increasing after deconjugation or hydrolysis of caffeoylquinic acid, [27] hence this inverse relationship after treatment processes in our study. Hydroxycinnamic acids exhibits geometric isomerism, [23] and these artefactual changes during extraction result in the different isomers identified. The contents of 2-hydroxy-3-*O-β-*D-glucopyranosyl-benzoic acid followed the trend of caffeoylquinic acid with treatment type. Protocatechuic acid had minute concentrations in all four treatments and is reportedly sparingly soluble in water with a high melting point, [28] thus we attribute the low content to its sedimentation into the pellet after centrifugation.

#### Iridoids and phenolic apioglucoside

In general, heat extractions gave higher contents of IGs than the cold extractions in the order HNP>HP>CP>CNP (Fig 3B). HNP contained the largest amount of sweroside, acetylloganic acid isomers, and secoxyloganin than the other treatments, only morroniside did not show any significant change due to treatment. Hot-no-pectinase extractions and HP had a higher overall contribution to the IGs content, 35% and 29%, respectively. The only phenolic apioglucoside identified for *S*. *cocculoides* was kelampayoside A. It had higher contents after heat treatments, (Fig 3C). The high contents of IGs and kelampayoside A after heat treatment present an advantage in the maintenance of health beneficial compounds during the design of thermally processed *S*. *cocculoides* foods.

#### Flavonoids

Six flavonoids were identified in *S*. *cocculoides* juice with rutin (a quercetin glucoside) as the predominant flavonoid after CNP treatment (Fig 3D). Similar to caffeoylquinic acid, heat with or without enzyme in the aqueous environment had a detrimental effect on the content of rutin. In contrast, quercetin galactoside, quercetin and kaempferol showed no significant difference between CP and HP. For naringenin glucoside isomers, HP, HNP and CP treatments showed no significant difference (p > 0.05) and were higher than CNP treatments. Upon pectinase hydrolysis, rutin is degraded to its more stable aglycone quercetin (by deglycosylation) and the disaccharide rutinose, thus a strong negative correlation with rutin and quercetin (R = -0.84) was realised. Commercial pectinases preparations often display secondary enzymatic activities that can change the profile of PCs, [29] hence the degradation of rutin to quercetin is most probably due to the presence of glycosidase in the enzyme preparation. Additionally, rutin is degraded by the heat because of the reactive oxygen species: (^.^O^-^_2,_ HO^-^_2_^.^ and HO^.-^), [30] which were also probably increased by the surface area exposed to water and oxygen. Overall, we expect the biological effect of flavonoid aglycones to be more relevant for thermal processing of *S*. *cocculoides* juice due to their stability during heat treatments.

### Multivariate data analysis

**Fig 4:**
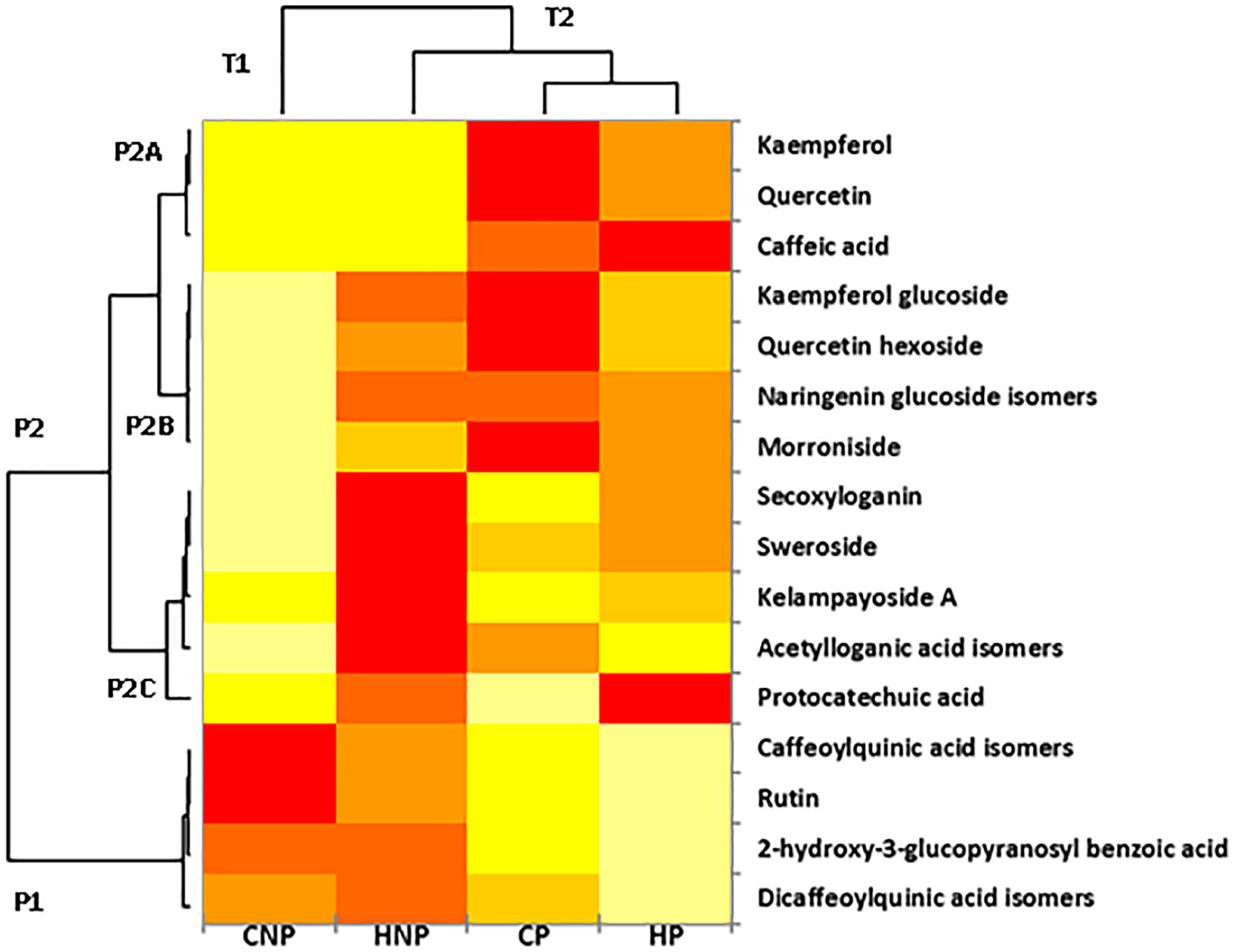
Heatmap calculated from quantitative data of polyphenol concentrations in the four different treatments (CNP, HNP, CP and HP). **The color scale moves from yellow to red with increased content. The clusters P1, P2, T1 and T2 refer to the polyphenol classes and to the technological treatments, respectively.** CNP: Cold water extraction without pectinase, HNP: Hot water extraction without pectinase, HP: Hot water extraction with pectinase, CP: Cold water extraction with pectinase.

Fig 4 shows the heat map as constructed for the simultaneous analysis of variables and samples clustering in a synthetic way. Cluster analysis quantified the degree of similarity between PCs as well as the four treatments, by calculating the distance between the possible pairs of molecules and treatments as described by Ward [18]. The hierarchical clustering grouped treatments into cluster T1 and T2, corresponding to the group with and without pectinase respectively, while the polyphenols were clustered into two principal groups: P1 and P2. Cluster P1 included esterified compounds i.e caffeoylquinic acid, dicaffeoylquinic acid isomers, rutin and 2-hydroxy-3-*O-β-*D-glucopyranosyl benzoic acid. These compounds were efficiently extracted in non- pectinase treatments, while the presence of pectinase caused degradation of the ester linkages resulting in overall lower concentration of the initially characterized compounds. Interestingly, temperature further degraded rutin and caffeoylquinic acid isomers, while 2-hydroxy-3-*O-β-*D-glucopyranosyl benzoic acid and dicaffeoylquinic acid isomers were scarcely influenced by temperature. Cluster P2 contained three subgroups: (P2C) IGs and kelampayoside; (P2B) flavonoid glycosides and morroniside, and (P2A) flavonoid aglycones and CA. Groups, P2A and P2B, defined the molecules positively influenced by the presence of pectinase in cold treatments which promoted their extraction. A well-defined behaviour was also apparent for IGs: the best performance was obtained at HNP, with the exception of morroniside, which was efficiently extracted with CP and for this reason very close to the flavonoid glycosides.

**Fig 5:**
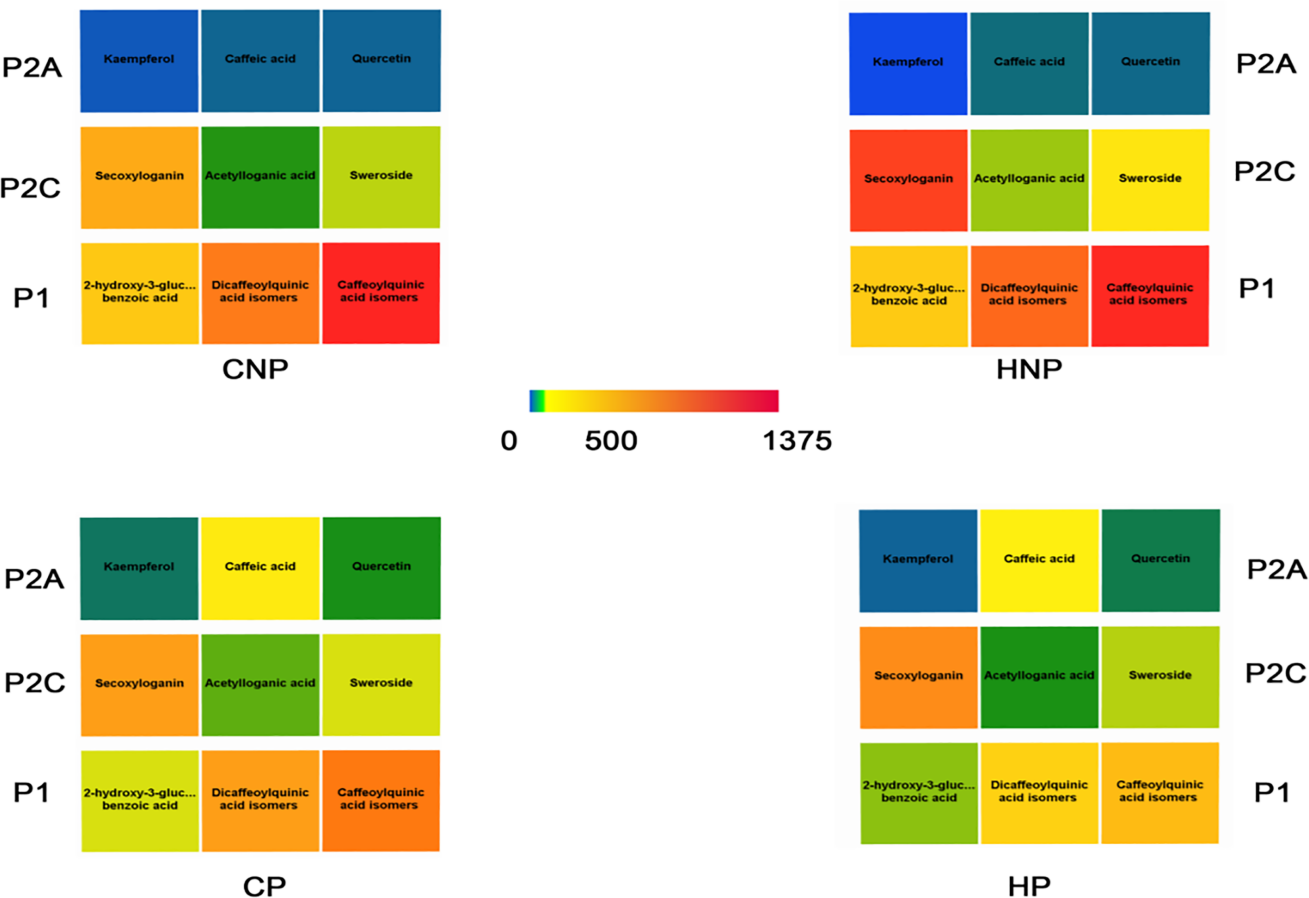
FancyTiles schema for the four extraction methods of *Strychnos cocculoides*. The values of the scale are reported in *μ*g/g DW while P2A, P2C and P1 report the clustering highlighted by the dendrogram in Fig 4.

The clustering outcomes were used to construct FancyTiles in Fig 5. This approach combined with the quantification of the target analytes, summarises the main quantitative differences between the four treatments in a spectrum-inverted chromatic scale, giving an immediate snapshot of the effects of treatments on PCs. Apart from the use of a logarithmic scale, quantitative tiles were presented by previous authors for tea seed oils combining simplified outputs with mass spectrometry quantification [31]. Nine compounds were divided into three categories (P2A, P2C and P1) according to the hierarchical clustering in Fig 4. For each treatment a specific tile was created by using fixed ranges. For P2A, the colour moved from blue to yellow and green, for pectinase to no-pectinase for CA and quercetin, respectively, while kaempferol remained close to the blue colour. Pectinase influenced the hydrolysis of esterified compounds in P1 that consequently moved from red/orange tonality to yellow green. Hot-no-pectinase promoted the efficient extraction of secoxyloganin and sweroside in P2C, which showed red and yellow tiles, from the orange and green tiles.

The data about the modification of PCs profile of the juice obtained with different extraction procedures highlight the relevance of processing in quality of the final product. At this stage it is not established if it is advisable to have more glycosides, aglycon, dicaffeoylquinic isomers or simple phenolic acids, however the knowledge of these details might have important consequence of food design in the era of personalized nutrition.

### Antioxidant activity

**Fig 6:**
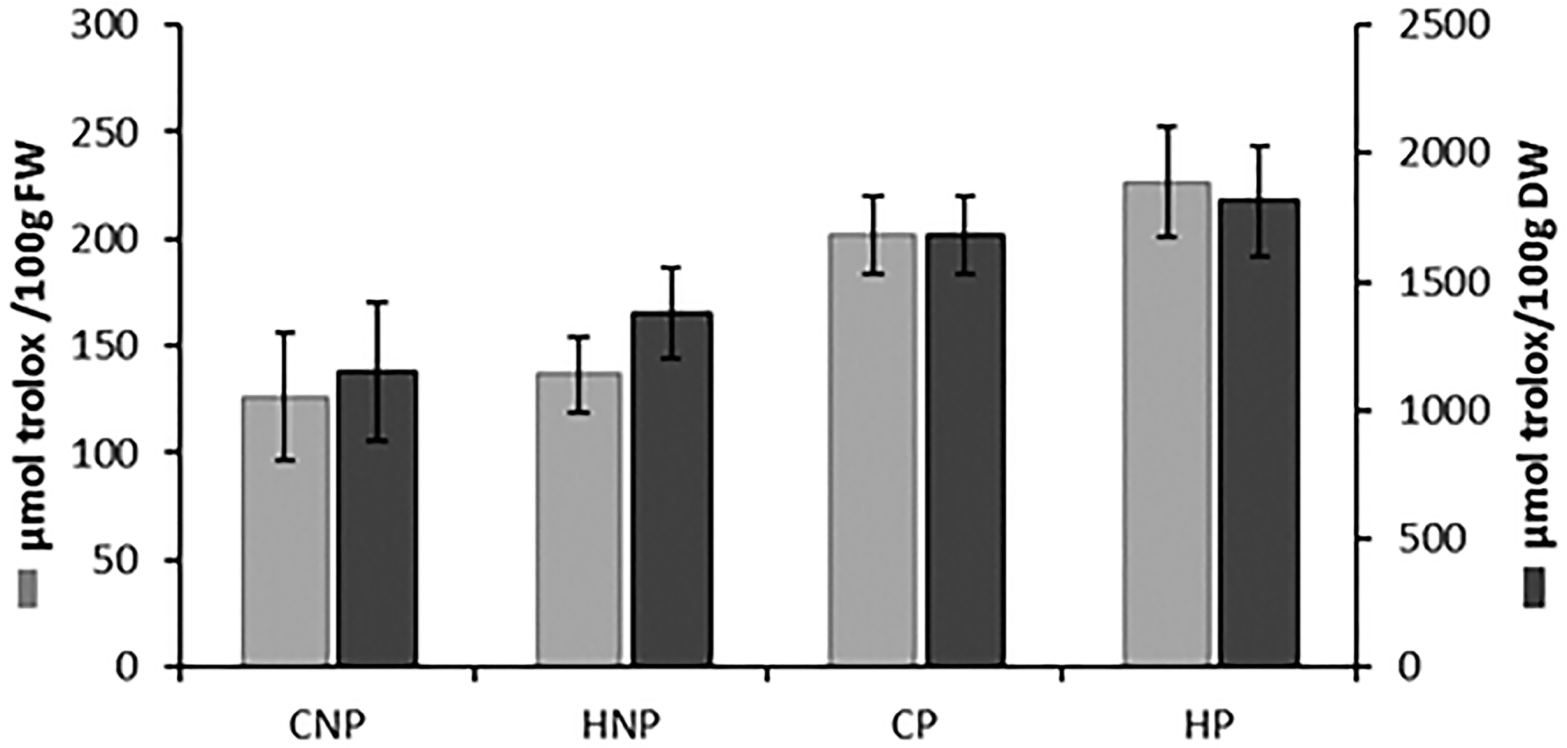
TEAC of *Strychnos cocculoides* juice. **Data are expressed as *μ*mol trolox/ 100 g ± standard error.** CNP: Cold water extraction without pectinase, HNP: Hot water extraction without pectinase, HP: Hot water extraction with pectinase, CP: Cold water extraction with pectinase.

Enzymatic treatments had higher AOA than the non-enzyme treatments due to the enhanced solubility of bioactive compounds, Fig 6. A statistically significant correlation existed between CA and AOA (*R* = 0.989; p = 0.01) and positive correlations for quercetin and AOA (*R* = 0.885) and kaempferol and AOA (*R* = 0.802) were also found. Inverse relationships were observed for 2-hydroxy-3-*O-β-*D-glucopyranosyl-benzoic acid (*R* = -0.997; p = 0.003), rutin (*R* = -0.975; p = 0.02), dicaffeoylqinic acid isomers (*R* = -0.85) and caffeoylqunic acid isomers (*R* = -0.938) with AOA.

The obtained correlations corroborate similar effects on the PCs contents of each treatment and the contribution to AOA at different treatments. Caffeic esters at higher concentrations are susceptible to oxidation, [23] thus a negative correlation with AOA follows as was expected at HP. Low AOA by CNP was probably caused by the release and action of cytoplasmic polyphenol oxidases responsible for PCs enzymatic oxidation [32]. Compounds not detected by MS/ MS have been reported when treatments at high temperatures and exposure times form antioxidant active nonphenolic compounds and phenol derivative compounds of higher AOA than their parent PCs [33]. Additionally, the radical scavenging capacity test is also not only sensitive to PCs but also other compounds such as organic acids, reducing sugars and synergistic effects between phenolics[34]. From the current findings, the presence of PCs such as flavonoid aglycones, which were relatively stable at HP, and their strong positive correlation with AOA, would warrant these compounds as contributory to the increased AOA at high temperatures. However, we cannot attribute AOA wholly to a single or class of PCs, and thus propose to investigate the synergistic effect with other different antioxidants in *S*. *cocculoides* pulp.

## Conclusions

Pectinase treatments macerated plant cell walls and helped to detach pulp from the outer endocarp of the fruit, providing a valuable tool to increase pulp yield and reduce waste. The secondary metabolites reported in *S*. *cocculoides* provide pharmacological properties, namely anti-inflammation, anti-cancer, antiviral and antimicrobial properties that benefit human health, [6, 8] improve shelf life and safety of food products from which they are processed. This demonstrates the potential for improved food security by increased utilization and value addition of *S*. *cocculoides* pulp for products consumed in Sub Saharan Africa where the fruit proliferates. The findings also open technological avenues for use by relevant stakeholders, such as research institutes, government and (Non-Governmental Organizations (NGOs), for diversification of juice processing that leads to a subsequent improved use of this and other underutilized indigenous fruits. However, considerations have to be made to suite consumer preference, taste and other intrinsic features such as endogenous enzymes and pectin content that could interfere with the outcome of enzymatic treatments. This still warrants further experimental work.

## Supporting information

S1 Fig Graphical abstract and observed pulp maceration from *S*. *cocculoides* fruit. (TIF)

S1 Table Overview and quantification of iridoid and phenolic compounds identified in *Strychnos cocculoides* juice extract. **Rt (retention time). The molecular ions were reported as [M-H]**^**-**^ **for negative ionization mode, [M+H]**^**+**^ **for positive ionization mode and [M+H]**^**+**^ for Na adduct.* Values are means ± SD n = 3. ND: Not Detected.
(DOC)
