# Supplementary figures and images for: Effect of heat and pectinase maceration on phenolic compounds and physicochemical quality of Strychnos cocculoides juice
Source: PLoS One. 2018 Aug 17;13(8):e0202415. doi: 10.1371/journal.pone.0202415 (PMC6097836; doi:10.1371/journal.pone.0202415)

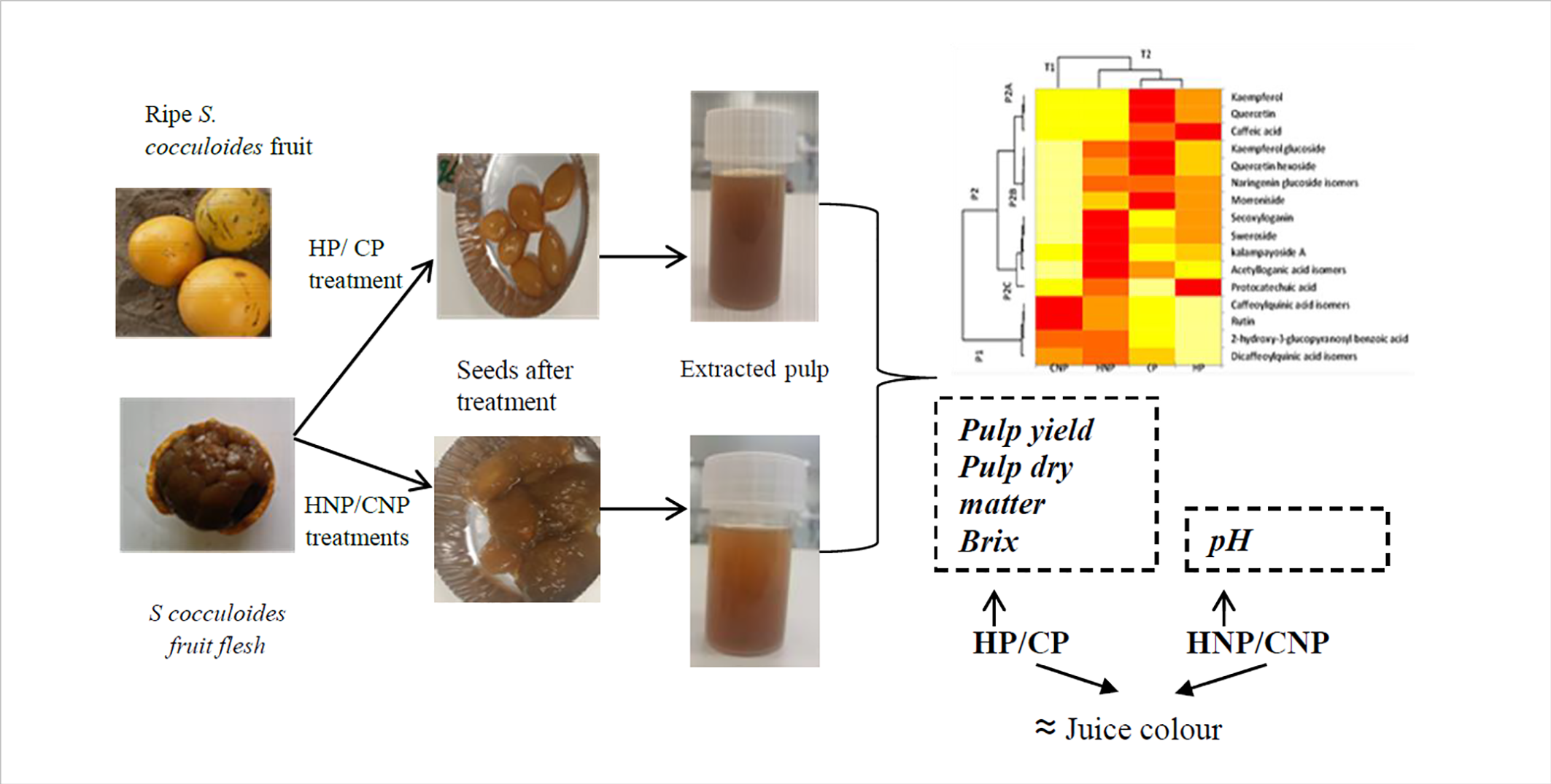

Supplement: S1 Fig — (TIF) [file pone.0202415.s001.tif]
