# Supplementary material for: Effect of heat and pectinase maceration on phenolic compounds and physicochemical quality of Strychnos cocculoides juice
Source: PLoS One. 2018 Aug 17;13(8):e0202415. doi: 10.1371/journal.pone.0202415 (PMC6097836; doi:10.1371/journal.pone.0202415)
Supplement: S1 Table — Rt (retention time). The molecular ions were reported as [M-H]- for negative ionization mode, [M+H]+ for positive ionization mode and [M+H]+ for Na adduct.* Values are means ± SD n = 3. ND: Not Detected. (DOC) [file pone.0202415.s002.doc]

| Investigated compounds | Formula | Exact mass | Molecular ions | Rt (min) | Maceration treatments *^*^* | | | |
| --- | --- | --- | --- | --- | --- | --- | --- | --- |
| **Phenolic acids** |  |  |  |  | CNP | HNP | CP | HP |
| Dicaffeoylquinic acid | C_25_H_24_O_12_ | 515.11950 | [M-H] - | 13.6 | 414.58  ± 27.29 | 442.55  ± 14.32 | 387.28  ± 18.06 | 263.57  ± 0.99 |
| Caffeoylquinic acid | C_16_H_18_O_9_ | 353.08781 | [M-H] - | 6.5 | 1312.16  ± 41.36 | 1231.23  ± 3.99 | 1175.74  ± 49.03 | 1132.11  ± 38.34 |
| Caffeic acid | C_9_H_8_O_4_ | 179.03498 | [M-H] - | 6.6 | 0.39  ± 0.08 | 0.56  ± 0.04 | 131.97  ± 8.99 | 149.52  ± 1.63 |
| 2-hydroxy-3- *O*-β-D-glucopyranosyl benzoic | C_13_H_16_0_9_ | 315.07216 | [M-H] - | 3.8 | 146.36  ± 2.05 | 144.83  ± 4.92 | 99.22  ± 2.72 | 78.61  ± 0.34 |
| Protocatechuic acid | C_7_H_6_O_4_ | 153.01933 | [M-H] - | 3.07 | 0.30  ± 0.01 | 0.55  ± 0.13 | ND | 0.71  ± 0.04 |
| **Flavonoids** |  |  |  |  |  |  |  |  |
| Naringenin glucoside isomers | C_21_H_22_O_10_ | 433.11042 | [M-H] - | 14.3 | 0.18  ± 0.00 | 0.39  ± 0.01 | 0.40  ± 0.01 | 0.37  ± 0.01 |
| Kaempferol | C_15_H_10_O_6_ | 285.04046 | [M-H] - | 18.8 | 0.04  ± 0.01 | 0.02  ± 0.01 | 1.29  ± 0.13 | 0.68  ± 0.02 |
| Quercetin | C_15_H_10_O_7_ | 301.03538 | [M-H] - | 16.2 | 0.35  ± 0.07 | 0.45  ± 0.04 | 8.61  ± 0.70 | 5.92  ± 0.29 |
| Quercetin glucoside | C_12_H_20_ O_12_ | 463.0882 | [M-H] - | 11.7 | 3.59  ± 0.04 | 7.95  ± 0.05 | 9.04  ± 0.42 | 6.95  ± 0.01 |
| Kaempferol 7-*O*-glucoside | C_21_H_20_O_11_ | 447.09328 | [M-H] - | 13.0 | 1.09  ± 0.04 | 2.86  ± 0.05 | 3.18  ± 0.17 | 2.25  ± 0.06 |
| Quercetin 3-*O*-rutinoside | C_27_H_30_O_16_ | 609.14611 | [M-H] - | 11.3 | 20.90  ± 0.65 | 16.36  ± 0.12 | 10.50  ± 0.32 | 7.32  ± 0.30 |
| **Iridoids** |  |  |  |  |  |  |  |  |
| Acetyl loganic acid isomers | C_18_H_26_O_11_ | 417.14023 | [M-H] - | 6.9-8.3-8.7 | 8.85  ± 0.51 | 29.28  ± 2.34 | 22.04  ± 0.52 | 13.97  ± 1.1 |
| Sweroside | C_16_H_22_O_9_ | 359.13366 | [M+H]^+^ | 8.2 | 62.3  ± 2.91 | 122.93  ± 2.2 | 99.35  ± 6.85 | 101.21  ± 1.99 |
| Morroniside | C_17_H_26_O_11_ | 429.1367 | [M+Na]^+^ | 6.2 | 43.81  ± 16.2 | 61.80  ± 10.53 | 75.83  ± 1.44 | 65.64  ± 11.02 |
| Secoxyloganin | C_17_H_24_O_11_ | 405.13914 | [M+H]^+^ | 5.5-6.3 | 189.58  ± 3.39 | 688.39  ± 5.31 | 390.07  ± 43.47 | 555.1  ± 2.76 |
| **Phenolic apioglucoside** |  |  |  |  |  |  |  |  |
| Kelampayoside A | C_20_H_30_O_13_ | 501.1579 | [M+Na]^+^ | 7.2 | 22.38  ± 1.5 | 34.15  ± 0.75 | 23.07  ± 0.01 | 27.67  ± 0.48 |

^*^ Values are means ± SD n=3. ND: Not Detected
